# Same Society, Meeting Sept. 27

**Published:** 1875-11

**Authors:** E. Warren Sawyer


### CHICAGO SOCIETY OF PHYSICIANS AND SURGEONS.

*Regular Meeting, Sept.* 27, 1875.

(Reported by E. WARREN SAWYER, M.D.)

Vice-President Hamill, in the chair.

The report of the Section on Materia Medica and Therapeutics being called for, it was presented and read by Dr. P. S. Hayes.

Dr. A. Reeves Jackson reported the following case:

### CASE OF STERNO-MASTOID TUMOR.

Mrs. T., a primipara, aged 24 years, was delivered of a female child, Nov. 25, 1874. The labor was a lingering one and very severe, delivery being finally accomplished with forceps. On the 18th of December, following—

twenty-four days after confinement—the mother called my attention to a swelling which had appeared on the right side of the infant's neck. The site of the enlargement was the sterno-cleido-mastoid muscle, which had assumed a sigmoid shape, the upper or mastoid extremity curving forward and the sternal end backward. The middle and lower thirds of the muscle were more swollen than the upper third. Throughout its entire length it was indurated, and, under tolerably firm pressure, it seemed painless. The muscle could be moved laterally or pressed inwards, although the degree of mobility was somewhat less than natural. The skin covering the part was not altered and was freely movable over the surface of the swelling. There was, apparently, no impairment of motion in the muscles of the neck, for the child moved its head without constraint in any direction.

I advised the daily application of tincture of iodine to the part, and this treatment was continued for a few days without noticeable effect. I then prescribed inunction with mercurial ointment, twice a day. The swelling slowly subsided, and the muscle gradually resumed its normal shape and size, so that at the end of about six months, all trace of the disease had disappeared. The ointment was used nearly four months. It produced no constitutional symptoms, and, I think, had also no influence locally. Indeed, the mother thought the growth was reduced more rapidly after she ceased using it.

At a meeting of the London Pathological Society, held Nov. 17, 1874, (*Medical Times and Gazette*, London, Nov. 28, 1874), Dr. Frederick Taylor brought forward a case of induration of the sterno-mastoid muscle in an infant.

The subject of the disease was first seen at the age of four weeks, when, in addition to a syphilitic eruption, it presented the condition known as tumor of the sterno-mastoid.

There was an unquestionable history of syphilis in the parents. While improving under mercurial treatment, the child died very soon of broncho-pneumonia.

Post-mortem, a hardened nodular mass could be felt under the skin occupying the lower half of the muscle, and freely movable subcutaneously. On dissection, the muscle was found perfectly free from the surrounding tissues. The sternal end chiefly was indurated; and the induration, to the naked eye, presented the characters of muscular tissue only. Microscopically, however, the more dense portions appeared to consist almost entirely of white fibrous tissue; and this element passed upwards beyond the limits of induration, separating and isolating the muscular fibres. There was an abnormal development of fibrous tissue in the clavicular portion also of the muscle. In no situation was there any young cell-growth to be discovered; all was fibrillar.

The exhibitor said that among the several explanations offered of the origin of these tumors, was that of their development by simple hypertrophy, and that by inflammation. Mr. Thomas Smith had lately considered that they might be due to injuries suffered during birth; other pathologists considered them syphilitic in their nature.

In the present instance there was certainly syphilis; and the child was born by the breech, with the hands over the head. But neither did the dissection reveal hæmorrhage or adhesions around the muscle, nor did the microscopical examination yield evidence of inflammation. Nothing definite of a positive kind had therefore been made out.

It was another question what becomes of these tumors. Observers who declare that they disappear, have perhaps not watched them sufficiently long. It was possible that some cases of imperfect development of one side of the neck might have this origin.

Mr. Arnott said that this was, perhaps, the first record published of the histology of the sterno-mastoid tumor. As to its cause, of many cases which he had seen, there had been unnatural delivery in one only, and in not one had there been syphilis. Such tumors were not seen, as a rule, for a fortnight after birth, probably because the mother does not usually wash the child before that time.

Mr. Clement Lucas related the case of a child with a tumor of the buccinator muscle, which appeared shortly after birth and disappeared under treatment by mercurial inunction. At the age of six months the child presented syphilitic symptoms.

Dr. Taylor remarked that Mr. Thomas Smith mentions one case of sterno-mastoid tumor which was observed immediately after birth.

The President (Sir Wm. Jenner), believed that the non-discovery of the tumor until a fortnight after birth, was to be explained by the custom of intrusting the dressing of the infant to the nurse.

The case related by Dr. Taylor so nearly resembles the one observed by me in most of its features that I cannot but regard the two as identical in character. But, while in the former there was abundant evidence of the presence of a syphilitic taint, in the latter there was none, both parents having been, and still being healthy. The child, too, now over ten months old, has been free from any skin disease and remarkably healthy. Mr. Arnott in the discussion alluded to, stated that he had seen "many cases," and that there had been no history of syphilis in any of them. So far, therefore, as we have any evidence upon the subject, I think we may regard the presence of syphilis, when it occurs, as accidental and not as an etiological factor.

Histologically, these enlarged indurations seemed to occupy a doubtful position. Being confined wholly to the muscle, they must, probably, be regarded as either hypertrophic or inflammatory in character. They can hardly be considered inflammatory, I think. Dr. Taylor says, positively, that in the case examined by him there were no evidences of inflammation. If the part were acutely inflamed there would be present other symptoms

—pain, impaired motion, increased heat, and a tendency to suppuration. These were all absent. If, on the other hand, the inflammation were of the chronic form, we would have, after the disease had passed beyond the stage of hyperæmia, the formation of an interstitial germinal tissue—the exudation of authors; and where from this there results "induration," the muscular fibres perish entirely, and there remains only a narrow ribbon-like stripe, where there was formerly a full muscular belly.*

As regards their being hypertrophic, Rindfleisch says that true hypertrophy of muscular tissue occurs in a marked manner only in the heart muscle; that although the voluntary muscles may become large and more vigorous by exercise, such hypertrophies cannot, at present, be demonstrated anatomically.

There is another form of enlargement of muscular tissue, denominated by Rindfleisch, pseudo-hypertrophy, which consists essentially in the permeation of the muscles with fat; and it is possible that it is in this pathological category that the disease under consideration belongs. Here, according to the authority just named, "the muscular fibres do not appear diminished, but only forced apart; none touches its neighbor because it is separated from it by a row of interstitial fat-cells."

---

### ROCK RIVER MEDICAL SOCIETY.

*Meeting, September* 27, 1875.

(Reported by F. E. SHERMAN, M.D., Secretary.)

The Rock River Medical Society met pursuant to adjournment, at New Cassell, Fond du Lac county, Wis., and was called to order at 10.30 A.M. President Senn in the chair.

Dr. Marston, of New Cassell, reported an interesting

* Rindfleisch, Text-Book of Pathological Anatomy, p. 676.

